# Outcomes of Pediatric Tracheostomy: The Impact of Body Weight on Complications

**DOI:** 10.3390/jcm15135015

**Published:** 2026-06-27

**Authors:** Ferhat Sarı, Elif Sari, Dastan Temirbekov, Aynur Aliyeva

**Affiliations:** 1 Department of Pediatrics, Division of Pediatric Intensive Care, Faculty of Medicine, Istanbul Aydın University, Istanbul 34295, Turkey; ferhatsari_dr@hotmail.com; 2 Department of Otorhinolaryngology-Head and Neck Surgery, Istanbul Aydın University, VM Medikal Park, Florya Hospital, Istanbul 34295, Turkey; ctf_elif@hotmail.com (E.S.); dasekeeee@gmail.com (D.T.); 3 Department of Surgery, Denver Health Medical Center, Denver, CO 80204, USA; 4 Neuroscience Doctoral Program, Yeditepe University, Istanbul 34755, Turkey

**Keywords:** pediatric tracheostomy, weight-based risk stratification, cannula-related complications, intensive care outcomes, predictive modeling

## Abstract

**Background**: Pediatric tracheostomy is a critical airway intervention associated with technical challenges and procedure-related complications, particularly in infants and small children. Body weight may influence perioperative risk, yet weight-based outcome data remain limited. **Objective**: The study aimed to evaluate the impact of body weight on acute complications and the clinical course following pediatric tracheostomy, with a particular focus on low-weight infants. **Methods**: This retrospective cohort study included 39 pediatric patients who underwent tracheostomy in a tertiary pediatric intensive care unit. Patients were stratified by body weight into three groups. Demographic data, indications, procedural characteristics, acute complications, and clinical outcomes were analyzed. **Results**: Acute complications occurred in 7 patients, most commonly cannula-related events. Complication rates were higher in patients < 5 kg. Patients < 5 kg had a longer mean hospital stay than the heavier groups. **Conclusions**: Low body weight showed a clinically relevant trend toward higher technical risk and prolonged hospitalization following pediatric tracheostomy, although statistical significance was not reached in this cohort. Weight-based procedural planning may support safer postoperative management in this vulnerable population.

## 1. Introduction

Pediatric tracheostomy is a life-saving intervention for children requiring prolonged airway support due to neurological impairment, chronic lung disease, neuromuscular disorders, or upper airway obstruction [1,2,3]. Despite advances in pediatric critical care, tracheostomy in children remains technically challenging and is associated with procedure-related complications, prolonged hospitalization, and significant caregiver burden [4,5]. Unlike adult populations, pediatric patients exhibit wide heterogeneity in age, anatomy, and body weight, all of which may influence procedural complexity and postoperative outcomes [6,7].

Body weight is a particularly relevant but under-studied factor in pediatric tracheostomy [8,9]. Infants and young children have shorter tracheal length, reduced airway diameter, and limited tolerance for cannula mismatch, which may predispose them to cannula-related complications such as ventilation difficulties, accidental decannulation, atelectasis, and entry-site complications. In routine clinical practice, low-weight patients often require individualized cannula selection, closer monitoring, and technical adjustments; however, objective data supporting weight-based risk stratification remain limited [10,11].

Previous pediatric tracheostomy studies have primarily focused on indications, overall complication rates, or long-term survival, with mortality largely reflecting underlying disease rather than tracheostomy itself [12,13]. Few studies have systematically evaluated the impact of body weight on acute technical complications and perioperative clinical course in a real-world pediatric intensive care unit setting [14,15]. Moreover, the relationship between weight, tracheostomy timing, duration of mechanical ventilation, and hospital length of stay remains unclear [16,17].

Understanding whether low body weight constitutes a distinct technical risk category has direct clinical implications for surgical planning, cannula selection, postoperative surveillance, and family counseling. Identifying weight-associated trends in complications and outcomes may help refine procedural strategies and optimize care in vulnerable pediatric populations [15,16,17,18,19].

Although previous pediatric tracheostomy studies have reported indications, complication rates, and long-term outcomes, limited evidence specifically evaluates body weight as a technical risk factor for acute cannula-related complications in the pediatric intensive care setting. The novelty of the present study lies in its weight-stratified assessment of perioperative complications, hospitalization, ventilatory course, and exploratory clinical risk features in a real-world PICU cohort, with particular attention to infants weighing < 5 kg.

The primary objective of this study was to evaluate whether low body weight (<5 kg) is associated with an increased risk of acute cannula-related complications following pediatric tracheostomy. Secondary objectives included assessing the association between body weight and key clinical outcomes, including total hospital length of stay, duration of mechanical ventilation after tracheostomy, decannulation, and in-hospital mortality. We further explored correlations between age, weight, timing of tracheostomy, and postoperative ventilatory burden. The study was based on the hypothesis that children weighing < 5 kg would show higher rates of cannula-related complications and a more prolonged clinical course than heavier patients.

## 2. Materials and Methods

### 2.1. Study Design and Setting

This study was designed as a retrospective cohort analysis conducted in a tertiary-level pediatric intensive care unit (PICU). Medical records of pediatric patients who underwent tracheostomy during the study period were reviewed. The study reflects real-world clinical practice at a tertiary referral center that manages complex pediatric airway and critical care cases.

### 2.2. Study Population

A total of 39 pediatric patients who underwent surgical tracheostomy were included in the analysis. Eligibility criteria were defined as follows:

Inclusion criteria:

- Pediatric patients who underwent surgical tracheostomy during PICU admission.
- Availability of complete demographic, clinical, procedural, and outcome data in institutional medical records.
- Documented in-hospital follow-up until discharge or death.

Exclusion criteria:

- Incomplete documentation regarding the perioperative course.
- Missing postoperative outcome data.
- Insufficient medical record information for weight-based stratification or complication assessment.

All patients were followed during hospitalization, and postoperative outcomes were assessed until discharge or death. The study was conducted in accordance with the principles of the Declaration of Helsinki. Approval was obtained from the Istanbul Aydin University (Approval No. = 131/2024 on 11 December 2024) ethics committee before data collection.

### 2.3. Data Collection and Variables

**Figure 1:**
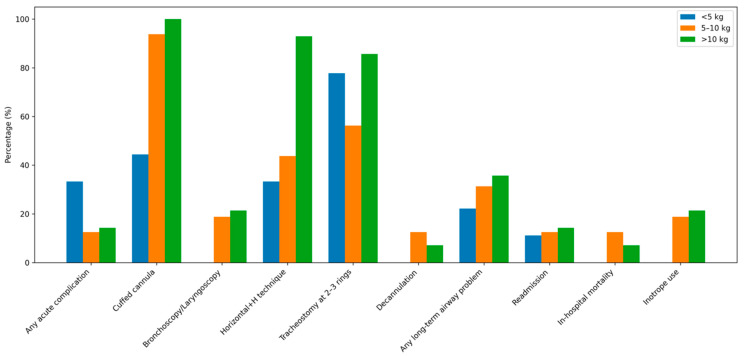
Weight Stratified Clinical Outcomes.

Demographic variables included age in months, recorded gender, and body weight in kilograms at the time of tracheostomy. Patients were stratified into three predefined weight groups based on clinical relevance and prior pediatric airway literature: <5 kg, 5–10 kg, and >10 kg. Primary indications for tracheostomy were categorized according to underlying disease, including hypoxic–ischemic encephalopathy, bronchopulmonary dysplasia with prolonged intubation, neuromuscular and neurologic diseases, metabolic disorders, upper airway obstruction, sepsis or acute respiratory distress syndrome, post-transplant Acute Respiratory Distress Syndrome(ARDS), cerebral palsy, terminal brain tumors, and Guillain–Barré syndrome (Table 1, Table 2 and Table 3). Figure 1 visually summarizes the weight-stratified distribution of clinical parameters and complication patterns, highlighting the higher numerical burden of acute complications and longer hospitalization observed in the <5 kg group.

Procedural variables included tracheostomy technique (modified “horizontal “H” and modified Bjork flap tracheostomy) [20], tracheostomy level (between second–third, third–fourth, or fourth–fifth tracheal rings), cannula diameter and length, cuffed versus non-cuffed cannula use, and performance of bronchoscopy or laryngoscopy during the perioperative period. Postoperative management variables included the duration of mechanical ventilation before and after tracheostomy, the interval from ICU admission to tracheostomy, and the total hospital length of stay.

### 2.4. Outcome Definitions

The primary endpoint was the occurrence of acute complications following tracheostomy. Acute complications were defined as any of the following events occurring during hospitalization: cannula-related complications (including cannula length mismatch, ventilation difficulty, or early accidental decannulation), cannula entry site infection, bleeding, or atelectasis. Cannula-related complications were additionally evaluated as a separate subgroup given their technical relevance, particularly in low-weight patients.

Cannula entry-site infection was defined retrospectively as documentation of local inflammatory findings at the tracheostomy site, including erythema, swelling, purulent discharge, a positive microbiological culture when available, or the need for topical or systemic antimicrobial treatment, as recorded in the medical chart. Long-term airway infection was defined as an infection documented during follow-up after the acute postoperative period. Because this was a retrospective study, infection classification was based on clinical documentation, and microbiological culture confirmation was not available or required in all cases.

Secondary endpoints included total hospital length of stay, duration of mechanical ventilation after tracheostomy, achievement of decannulation, tracheostomy-related readmission, long-term airway complications, in-hospital mortality, and need for inotropic support. Long-term airway problems were categorized as none, tracheal stenosis, bleeding, granulation tissue formation, or infection.

### 2.5. Exploratory Risk Stratification

An exploratory clinical risk stratification framework was constructed to describe patterns associated with tracheostomy-related complications rather than to establish a validated predictive model. Binary clinical features were selected based on clinical relevance and observed trends in this cohort: body weight < 5 kg, ICU-to-tracheostomy interval > 30 days, cannula diameter < 4 mm, and absence of a modified Björk flap technique. For descriptive purposes, each feature was assigned 1 point, yielding a total exploratory score ranging from 0 to 4. Patients were categorized as low risk (0–1 points), moderate risk (2–3 points), or high risk (4 points). This framework was applied retrospectively to all patients in the cohort and should be interpreted only as a hypothesis-generating clinical observation, requiring external validation in larger multicenter studies (Table 4).

### 2.6. Statistical Analysis

Continuous variables were summarized as mean ± standard deviation with ranges, while categorical variables were expressed as counts and percentages. Normality of continuous variables was assessed visually and analytically, and non-parametric methods were applied when appropriate. Group comparisons by weight category (<5 kg, 5–10 kg, >10 kg) were performed to evaluate differences in complication rates and clinical outcomes.

For categorical outcomes, comparisons between groups were conducted using Fisher’s exact test or chi-square test, depending on cell counts. The primary comparison focused on patients < 5 kg versus ≥5 kg patients, while a secondary three-group comparison was also performed. Continuous variables across weight groups were compared using the Kruskal–Wallis test due to non-normal distribution. Effect size for hospital length of stay comparisons was estimated using Cliff’s delta to assess the magnitude of differences independent of statistical significance.

Correlation analyses were performed using Spearman’s rank correlation coefficient to assess associations between age, weight, ICU-to-tracheostomy interval, and clinical course variables, including total hospital stay and duration of mechanical ventilation after tracheostomy. Exploratory univariable logistic regression analyses were conducted to estimate the association between low body weight (<5 kg) and risk of acute and cannula-related complications. Odds ratios with 95% confidence intervals were reported. Statistical significance was defined as a two-sided *p*-value <0.05. All analyses were performed using standard statistical software.

## 3. Results

### 3.1. Patient Demographics and Baseline Characteristics

A total of 39 pediatric patients undergoing tracheostomy were included in the analysis. The cohort consisted of 19 male and 20 female patients, with a mean age of 39.5 ± 50.3 months (range, 2–176 months) and a mean body weight of 14.6 ± 14.8 kg (range, 3.3–63 kg). Based on body weight at the time of tracheostomy, patients were stratified into three groups: <5 kg (*n* = 9, 23.1%), 5–10 kg (*n* = 16, 41.0%), and >10 kg (*n* = 14, 35.9%) (Table 1, Table 2 and Table 3).

The most frequent primary indication for tracheostomy was hypoxic–ischemic encephalopathy, accounting for 9 patients (23.1%), followed by bronchopulmonary dysplasia with prolonged intubation in 6 patients (15.4%) and spinal muscular atrophy or other neurologic diseases in 5 patients (12.8%). Metabolic diseases were observed in 4 patients (10.3%), while upper airway obstruction and other neuromuscular disorders each accounted for 3 patients (7.7%). Less common indications included sepsis or ARDS, post-transplant ARDS, cerebral palsy, terminal brain tumors, and Guillain–Barré syndrome The demographic characteristics, primary indications, procedural variables, and overall clinical outcomes of the study cohort are summarized in Table 1.

### 3.2. Procedural Characteristics and Perioperative Outcomes

Tracheostomy was performed using a modified Björk flap approach in 6 (15.4%) patients for long-term ventilation, and a Horizontal “H” approach in 33 (84.5%) patients. The tracheostomy was most frequently placed between the second and third tracheal rings (71.8%). A cuffed cannula was used in the majority of patients (87.2%), whereas 12.8% received a non-cuffed cannula. Bronchoscopy or laryngoscopy was performed perioperatively in 6 patients (15.4%). Decannulation was achieved in 3 patients (7.7%) during follow-up (Table 1).

#### Surgical Technique

**Figure 2:**
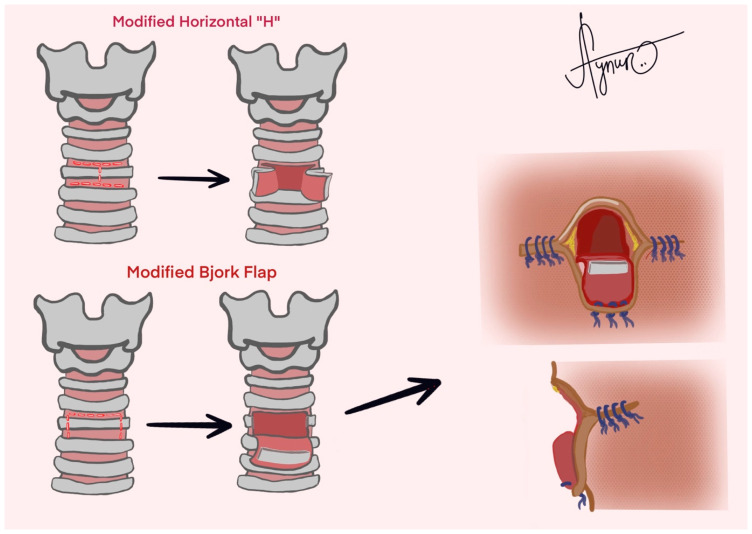
Modified pediatric tracheostomy techniques used in the study. Schematic illustration of the two cartilage-sparing tracheostomy techniques applied in this cohort: (**Up**) modified horizontal “H” tracheostomy, performed with a limited horizontal incision involving a single tracheal cartilage ring, typically between the second and third tracheal rings, incorporating the adjacent intercartilaginous membranes; and (**Down**) modified Björk flap tracheostomy, used selectively in patients requiring anticipated long-term mechanical ventilation, created by partial resection of a single tracheal cartilage ring to enhance cannula stability while preserving tracheal framework integrity.

All tracheostomy procedures were performed by an experienced otolaryngology team using a standardized open technique, with modifications tailored to pediatric airway anatomy and anticipated ventilation duration. The modified horizontal “H” incision and modified Björk flap techniques used in this cohort are schematically illustrated in Figure 2.

1. In most patients, a modified horizontal “H” tracheostomy was performed. This modification differed from the classical H incision by limiting the tracheal opening to a small horizontal incision involving a single tracheal cartilage ring only. The incision was performed primarily between the second and third tracheal rings and, in selected cases, between the third and fourth rings, incorporating the intercartilaginous membranes above and below the involved ring. No resection of multiple cartilage rings was performed, resulting in a minimal and controlled tracheal opening designed to reduce structural disruption in small pediatric airways.
2. In a selected subset of patients with an anticipated need for prolonged mechanical ventilation, a modified Björk flap technique was utilized. This approach was considered “modified” because it involved resection of only a single tracheal cartilage ring, rather than multiple rings as described in the conventional Björk flap. The flap was fashioned in a limited manner to provide stable cannula placement while preserving tracheal integrity. This technique was reserved for patients in whom long-term airway access was deemed necessary, based on underlying diagnosis and clinical course.

Across both techniques, the guiding surgical principle was to minimize cartilage resection, preserve the tracheal framework, and optimize cannula stability, particularly in low-weight infants with small, compliant airways.

### 3.3. Acute Complications and Long-Term Airway Outcomes

Acute complications occurred in 7 patients (17.9%), while 27 patients (69.2%) experienced no acute postoperative complications. The most common acute complication was atelectasis associated with cannula length mismatch (7.7%), followed by cannula entry site infection (5.1%), early accidental decannulation (2.6%), and bleeding (2.6%) (Table 1). Long-term airway problems were documented in 12 patients (30.8%), most commonly bleeding (15.4%) and infection (7.7%), while tracheal stenosis and granulation tissue formation were less frequent. Tracheostomy-related readmission occurred in 5 patients (12.8%), and in-hospital mortality was observed in 3 patients (7.7%). Inotropic support was required in 6 patients (15.4%).

### 3.4. Comparison of Clinical Outcomes by Weight Group

When stratified by weight, acute complications occurred in 3 of 9 patients (33.3%) in the <5 kg group, 2 of 16 patients (12.5%) in the 5–10 kg group, and 2 of 14 patients (14.3%) in the >10 kg group (Table 2, Figure 1). The <5 kg group had a numerically higher complication rate than heavier patients. Total hospital stay also differed across weight groups, with the longest mean hospitalization observed in patients weighing < 5 kg (50.3 ± 16.4 days), compared with 32.6 ± 20.0 days in the 5–10 kg group and 35.9 ± 14.5 days in the >10 kg group. Similarly, the ICU-to-tracheostomy interval and the duration of mechanical ventilation after tracheostomy tended to be longer in lower-weight patients. Weight-stratified comparisons of demographic, clinical, procedural, and outcome variables are presented in Table 2 and Table 3.

### 3.5. Correlation and Exploratory Analyses

Spearman correlation analysis demonstrated weak, non-significant negative correlations between age and total hospital stay (ρ = −0.229, *p* = 0.150) and between weight and total hospital stay (ρ = −0.254, *p* = 0.109). A statistically significant positive correlation was observed between the ICU-to-tracheostomy interval and duration of mechanical ventilation after tracheostomy (ρ = 0.322, *p* = 0.037), indicating that a longer delay to tracheostomy was associated with prolonged postoperative ventilatory support (Table 3). In univariable logistic regression analysis, body weight < 5 kg was associated with higher odds of any acute complication (OR 3.09, 95% CI 0.65–14.62, *p* = 0.156) and cannula-related complications (OR 4.64, 95% CI 0.92–23.48, *p* = 0.064), although these associations did not reach conventional statistical significance. The correlation analyses and exploratory univariable logistic regression results are summarized in Table 3.

To identify patients at higher risk of tracheostomy-related complications, a simple clinical prediction model was developed using four binary variables: weight < 5 kg, ICU-to-tracheostomy interval > 30 days, cannula diameter <4 mm, and absence of a modified Björk flap tracheostomy. Each variable was assigned one point, yielding a total risk score ranging from 0 to 4.

When applied to the cohort, patients were stratified into three risk categories: low (0–1 points), moderate (2–3 points), and high (4 points). Among patients classified as high risk, there was a noticeable trend toward more extended hospital stays and higher rates of cannula-related complications. However, statistical significance was limited by sample size. The risk score provided a practical framework to guide cannula selection and postoperative care intensity. This stratification model may help clinicians anticipate technical challenges and optimize early intervention for vulnerable patients, particularly those weighing under 5 kg (Table 4).

## 4. Discussion

The present study suggests that low body weight, particularly <5 kg, may be an important clinical factor associated with acute complications following pediatric tracheostomy, particularly cannula-related events. In this cohort, infants weighing < 5 kg had numerically higher rates of acute complications, longer hospital stays, and greater technical challenges than heavier infants; however, the exploratory logistic regression findings did not reach conventional statistical significance. Therefore, these results should be interpreted as clinically relevant trends rather than definitive evidence of an independent predictive relationship. Although statistical significance was not reached, infants weighing < 5 kg had higher complication rates, longer hospital stays, and greater technical challenges than heavier infants. These findings support body weight as a clinically relevant factor influencing perioperative risk in pediatric tracheostomy.

### 4.1. Comparison with Existing Literature

Reported complication rates after pediatric tracheostomy vary substantially across cohorts. In an extensive systematic review (49 studies; 1978–2020), the average overall complication rate was ~40%, with reported series ranging from 0% to 90%; tracheostomy-related mortality was reported up to 6% and was most attributed to cannula obstruction or accidental decannulation [21]. In that same review by Neto et al, pooled complication patterns across included studies were dominated by “technical/cannula-era” events (e.g., accidental decannulation 8.3%, cannula obstruction 8.0%), whereas bleeding (1.8%) and tracheal stenosis (2.6%) were less frequent. In our cohort (N = 39), the overall frequency of acute complications was 7/39 (17.9%). The <5 kg subgroup showed a higher acute complication proportion (3/9, 33.3%) compared with 5–10 kg (2/16, 12.5%) and >10 kg (2/14, 14.3%), consistent with literature suggesting that smaller and younger patients concentrate technical morbidity even when differences do not always reach statistical significance. The breadth of reported outcomes in the literature is also reflected in mortality variability across large datasets, as Dal’Astra et al. (5933) reported tracheostomy-related mortality of 1% and overall mortality of 12.4% in the review’s summary table, underscoring that overall outcomes are strongly influenced by baseline disease severity rather than procedure mechanics alone [12,20,21].

### 4.2. Pathophysiological and Technical Considerations

Low-weight infants present predictable anatomical constraints, including a shorter tracheal length, a smaller airway diameter, and a tighter tolerance for cannula length/curvature mismatch. In a Fenley et al. retrospective study of 171 pediatric patients, Fenley found that weight was the only statistically significant predictor of cross-sectional tracheal area and the most significant predictor of tracheal AP diameter, compared with airway area calculations from CT or MRI imaging [22]. The study findings also align with the Villarroel et al. publication, which demonstrated that an age-based formula provided accurate tracheostomy tube size predictions in 58% of patients, but when weight was incorporated into the formula, the prediction accuracy increased to 65% [23].

In the systematic review, the main fatal mechanisms were obstruction and accidental decannulation, again pointing to device–airway interaction rather than the skin incision itself. In our cohort, the distribution of complications and the weight-stratified pattern support the same logic: the <5 kg group had a numerically higher burden of acute complications (3/9, 33.3%), and the overall complication profile was dominated by cannula-related/technical events rather than catastrophic intraoperative injury. The observed trend aligns with broader evidence that younger/more minor children have higher susceptibility to complications due to airway size, secretion burden, and limited “margin for error” in cannula positioning, even in elective, experienced-center settings [21].

### 4.3. Practical Implications and Technical Strategies

In this cohort, technical complications were more frequent in infants weighing < 5 kg, occurring in 33.3% (3/9) compared with 12.5% (2/16) in the 5–10 kg group and 14.3% (2/14) in those >10 kg. These events were predominantly cannula- or airway-related rather than life-threatening. This pattern is consistent with the pediatric ICU series reported by Sachdev et al., in which overall complication rates ranged from 14% to 36% across perioperative and late periods, with non-catastrophic technical issues comprising the majority of events [3]. Their findings emphasize that pediatric tracheostomy morbidity is largely driven by airway management challenges rather than surgical failure. Together, these data support the need for a weight-adapted technical strategy in infants < 5 kg, including careful selection of cannula size and length, and closer postoperative surveillance.

In contrast to classical pediatric tracheotomy techniques that typically involve exposure and incision of 2–4 tracheal rings, the modified horizontal and limited Björk approaches used in our cohort intentionally preserved tracheal integrity by restricting the incision or flap to a single tracheal ring [9]. This conservative cartilage-sparing strategy aligns with contemporary surgical principles emphasizing reduced tracheal trauma and improved cannula stability in anatomically small and fragile pediatric airways. Compared with conventional pediatric tracheostomy techniques, our approach represents a restrained modification of both the horizontal incision and Björk flap concepts. While Kennedy et al. reported that classical Björk flap tracheostomy, involving wider cartilage resection, did not significantly alter complication or decannulation rates compared with window techniques, our modified Björk approach limits resection to a single tracheal cartilage and was selectively applied only in patients anticipated to require long-term ventilation, aiming to reduce airway trauma while maintaining cannula stability [22]. Similarly, Dal’Astra et al. emphasized that technique-related complications in children are largely technical rather than disease-driven; our limited horizontal–H modification, confined to the 2nd–3rd (or occasionally 3rd–4th) tracheal ring with minimal cartilage involvement, directly addresses this risk by adapting the surgical exposure to pediatric airway size rather than applying a uniform technique across weight groups [12].

### 4.4. Mortality and Clinical Outcomes

In-hospital mortality in our study was low and primarily related to underlying disease severity rather than tracheostomy-related complications. This aligns with the ICU-based pediatric tracheostomy literature, including the study by Sachdev et al., in which survival was largely determined by primary diagnosis and comorbidity burden rather than procedural factors. These findings reinforce the view that pediatric tracheostomy, when performed in experienced centers, is generally safe, with outcomes driven mainly by the child’s baseline condition rather than by technical complications.

### 4.5. Prediction Model for PICU Tracheostomy Outcomes

A key strength of the present study is the development of a simple, clinically applicable prediction model for tracheostomy-related complications in pediatric intensive care patients. Unlike most pediatric tracheostomy studies, which primarily report complication rates without structured risk stratification, our model integrates readily available perioperative variables, including body weight < 5 kg, ICU-to-tracheostomy interval > 30 days, small cannula diameter (<4 mm), and absence of a modified Björk flap. Although exploratory, this approach addresses an important gap in the literature, where predictive tools for tracheostomy-related risk in PICU populations remain scarce. The exploratory risk stratification approach is a strength of this manuscript, reflecting the growing role of artificial intelligence and machine-learning-based prediction models in individualized airway risk assessment, though it still requires validation in larger multicenter cohorts [24,25].

This concept aligns with the recently published 2025 study by Zoghi et al., who developed a validated predictive model for postoperative tracheostomy requirement in children undergoing medulloblastoma surgery, achieving an AUC of 0.845 using a simplified two-variable score based on brainstem involvement [26]. While Zoghi et al. focused on predicting the need for tracheostomy, our model uniquely targets post-tracheostomy technical complications, particularly in low-weight infants, and is applied across a heterogeneous PICU population. Together, these studies highlight a growing shift toward risk-based, data-driven decision support in pediatric airway management, underscoring the novelty and clinical relevance of incorporating prediction modeling into pediatric tracheostomy care [1,26].

### 4.6. Limitations

This study has several limitations, including its retrospective design, single-center setting, and relatively small sample size, which may limit statistical power and generalizability. The absence of multivariable modeling limits causal inference, and some clinically relevant factors, such as tracheal anatomy measurements, were unavailable. Nevertheless, the consistency of observed trends across multiple outcome measures strengthens the clinical relevance of the findings.

### 4.7. Clinical Implications

Although statistical significance was not reached, the observed direction of effect and weight-stratified complication pattern suggest that low body weight may be a clinically relevant technical risk modifier in pediatric tracheostomy. Awareness of this potential risk may help clinicians anticipate cannula-related challenges and apply closer postoperative surveillance in infants weighing < 5 kg. Because the exploratory regression findings did not reach conventional statistical significance, the observed weight-related differences should be interpreted as clinically relevant trends rather than definitive evidence of an independent predictive relationship.

### 4.8. Future Directions

Future studies should prospectively validate weight-based risk stratification for pediatric tracheostomy, particularly in infants weighing < 5 kg, using larger, multicenter cohorts. Refinement of the proposed prediction model with additional airway and perioperative variables may improve risk estimation and clinical applicability. Integration of machine-learning and artificial intelligence–based tools represents a promising direction for supporting individualized cannula selection, anticipating technical complications, and optimizing postoperative monitoring in the pediatric intensive care setting. Standardized, data-driven algorithms informed by such models may ultimately enhance safety and outcomes in vulnerable low-weight patients [27,28].

## 5. Conclusions

Pediatric tracheostomy is a safe and effective airway intervention; however, infants weighing < 5 kg showed a numerical trend toward higher rates of cannula-related technical complications and longer hospitalizations in this cohort. Because these findings did not reach conventional statistical significance, they should be interpreted cautiously and validated in larger multicenter studies. Targeted weight-based strategies focusing on cannula selection and postoperative monitoring may help improve safety in low-weight children.

## Figures and Tables

**Table 1:** Demographic and Clinical Characteristics of Pediatric Tracheostomy Patients (N = 39).

Parameter			Result
Age (months)			39.5 ± 50.3 (range: 2–176)
Weight (kg)			14.6 ± 14.8 (range: 3.3–63)
1	>5 kg		9 (23.1%)
2	5–10 kg		16 (41.0%)
5	<10 kg		14 (35.9%)
Gender (male/female)			19/20
Primary indication for tracheostomy, *n* (%)			
– Hypoxic–ischemic encephalopathy			9 (23.1%)
– Bronchopulmonary dysplasia with prolonged intubation			6 (15.4%)
– Spinal muscular atrophy/neurologic disease			5 (12.8%)
– Metabolic disease			4 (10.3%)
– Other neuromuscular diseases			3 (7.7%)
– Sepsis/ARDS			2 (5.1%)
– Post-transplant ARDS			2 (5.1%)
– Cerebral palsy			2 (5.1%)
– Terminal brain tumor			2 (5.1%)
– Guillain–Barré syndrome			1 (2.6%)
Acute complications, *n* (%)			
– None			27 (69.2%)
– Cannula entry site infection			2 (5.1%)
– Atelectasis with cannula length mismatch			3 (7.7%)
– Early accidental decannulation			1 (2.6%)
– Bleeding			1 (2.6%)
Total hospital stay (days)			38.1 ± 16.96 (range: ≥5–66)
ICU to tracheostomy interval (days)			26.8 ± 14.2 (range: 2–57)
Mechanical ventilation		before tracheostomy (days)	24.1 ± 13.9 (range: 2–51)
		after tracheostomy (days)	4.5 ± 4.4 (range: 0–21)
Cannula		diameter (mm)	4.4 ± 0.8 (range: 3–6)
		length (mm)	42.4 ± 2.4 (range: 39–48)
		Cuffed cannula, *n* (%)	34 (87.2%)
		Non-Cuffed cannula, *n* (%)	5 (12.8%)
Bronchoscopy/laryngoscopy performed, *n* (%)			6 (15.4%)
Tracheostomy type		Modified Horizontal H, *n* (%)	33(84.6%)
		Modified Bjork flap, *n* (%)	6 (15.4%)
Tracheostomy level		Between 2–3 rings, *n* (%)	28 (71.8%)
		Between 3–4 rings, *n* (%)	9 (23.1%)
		Between 4–5, *n* (%)	2 (5.1%)
Decannulation achieved, *n* (%)			3 (7.7%)
Long-term airway problems *n* (%)			
None			27 (69.2%)
Tracheal stenosis			1 (2.6%)
Bleeding			6 (15.4%)
Granulation tissue			2 (5.1%)
Infection			3 (7.7%)
Readmission related to tracheostomy, *n* (%)			5 (12.8%)
In-hospital mortality, *n* (%)			3 (7.7%)
Inotrop use, *n* (%)			6 (15.4%)

Note: Infections were classified according to retrospective clinical documentation; microbiological confirmation was not required in all cases. Values are presented as mean ± standard deviation with range or *n* (%), as appropriate. Descriptive statistics were used for overall cohort characteristics. ICU, intensive care unit; ARDS, acute respiratory distress syndrome.

**Table 2:** Comparison of Clinical Characteristics by Weight Group in Pediatric Tracheostomy Patients (N = 39).

Parameter	<5 kg (*n* = 9)	5–10 kg (*n* = 16)	>10 kg (*n* = 14)
Gender (male/female), *n*	3/6	7/9	9/5
Age (months)	3.8 ± 1.1 (2–5)	8.2 ± 1.4 (6–10)	92.6 ± 45.3 (13–176)
Weight (kg)	3.9 ± 0.6	7.4 ± 1.3	32.6 ± 16.9
Primary indication, *n* (% of total)			
Hypoxic–ischemic encephalopathy	4 (10.3%)	5 (12.8%)	0
Bronchopulmonary dysplasia/chronic lung disease	3 (7.7%)	2 (5.1%)	1 (2.6%)
Spinal muscular atrophy/neurologic disease	1 (2.6%)	4 (10.3%)	0
Metabolic disease	0	1 (2.6%)	3 (7.7%)
Upper airway obstruction	0	2 (5.1%)	1 (2.6%)
Other neuromuscular diseases	0	0	3 (7.7%)
Cerebral palsy	0	0	2 (5.1%)
Sepsis/ARDS	0	1 (2.6%)	1 (2.6%)
Post-transplant ARDS	0	1 (2.6%)	1 (2.6%)
Terminal brain tumor	0	0	2 (5.1%)
Guillain–Barré syndrome	0	0	1 (2.6%)
Any acute complication, *n* (% of group)	3 (33.3%)	2 (12.5%)	2 (14.3%)
Total hospital stay (days)	50.3 ± 16.4	32.6 ± 20.0	35.9 ± 14.5
ICU to tracheostomy (days)	37.0 ± 15.9	28.1 ± 17.0	21.1 ± 11.4
MV days before tracheostomy	26.8 ± 18.7	22.1 ± 16.7	19.5 ± 11.7
MV days after tracheostomy	7.8 ± 6.1	5.8 ± 5.3	2.5 ± 2.3
Cannula diameter (mm)	3.4 ± 0.4	4.2 ± 0.4	5.3 ± 0.5
Cannula length (mm)	39.8 ± 0.8	41.5 ± 0.8	44.9 ± 2.0
Cuffed cannula, *n* (% of group)	4 (44.4%)	15 (93.8%)	15 (100%)
Bronchoscopy/laryngoscopy, *n* (% of group)	0	3 (18.8%)	3 (21.4%)
Horizontal + H technique, *n* (% of group)	3 (33.3%)	7 (43.8%)	13 (92.9%)
Tracheostomy at 2–3 rings, *n* (% of group)	7 (77.8%)	9 (56.3%)	12 (85.7%)
Decannulation achieved, *n* (% of group)	0	2 (12.5%)	1 (7.1%)
Any long-term airway problem, *n* (% of group)	2 (22.2%)	5 (31.3%)	5 (35.7%)
Readmission, *n* (% of group)	1 (11.1%)	2 (12.5%)	2 (14.3%)
In-hospital mortality, *n* (% of group)	0	2 (12.5%)	1 (7.1%)
Inotrope use, *n* (% of group)	0	3 (18.8%)	3 (21.4%)

Note: Values are presented as mean ± standard deviation or *n* (%), as appropriate. Continuous variables were compared across weight groups using the Kruskal–Wallis test. Categorical variables were compared using chi-square or Fisher’s exact test, depending on cell counts. A two-sided *p*-value < 0.05 was considered statistically significant. ICU, intensive care unit; MV, mechanical ventilation; ARDS, acute respiratory distress syndrome.

**Table 3:** Correlation and Exploratory Regression Analyses.

Correlation Analysis: Age/Weight vs. Clinical Course			
Correlation	Spearman ρ	*p*-value	Interpretation
Age vs. Hospital Stay	−0.229	0.150	Not significant
Weight vs. Hospital Stay	−0.254	0.109	Not significant
ICU-to-Trach vs. MV Days After	+0.322	0.037	Significant
Logistic Regression			
Outcome	OR (95% CI)	*p*-value	Interpretation
Any acute complication	3.09 (0.65–14.62)	0.156	Not significant
Cannula-related complication	4.64 (0.92–23.48)	0.064	Borderline significant

Note: Correlations were assessed using Spearman’s rank correlation coefficient. Univariable logistic regression was used to estimate odds ratios with 95% confidence intervals for acute and cannula-related complications. A two-sided *p*-value < 0.05 was considered statistically significant. ICU, intensive care unit; MV, mechanical ventilation.

**Table 4:** Exploratory Clinical Risk Stratification Framework for Tracheostomy-Related Complications.

Predictor	Rationale
Weight < 5 kg	Strongest signal in all comparisons
ICU-to-tracheostomy > 30 days	Correlated with MV duration (ρ = 0.322)
Cannula diameter < 4 mm	Reflects anatomical challenge
No modified Björk flap	May increase technical risk and canul complications
For descriptive exploratory stratification, 1 point was assigned to each clinical feature: 0–1 points: Low-risk category 2–3 points: Moderate-risk category 4 points: High-risk category, suggesting the need for careful cannula selection and close postoperative monitoring	

Note: This framework was applied descriptively and was not statistically validated as a predictive model. It should be interpreted as an exploratory, hypothesis-generating clinical risk stratification framework. MV, mechanical ventilation;

## Data Availability

The datasets used and/or analyzed during the current study are available from the corresponding author on reasonable request.
